# Dehydration constrains thermoregulation and space use in lizards

**DOI:** 10.1371/journal.pone.0220384

**Published:** 2019-07-25

**Authors:** Marco Sannolo, Miguel Angel Carretero

**Affiliations:** 1 CIBIO, Research Centre in Biodiversity and Genetic Resources, InBIO, Universidade do Porto, Campus de Vairão, Rua Padre Armando Quintas, Vairão, Vila do Conde, Portugal; 2 Departamento de Biologia, Faculdade de Ciências da Universidade do Porto, R. Campo Alegre, Porto, Portugal; Universidade de São paulo, BRAZIL

## Abstract

Climate change is negatively affecting many species. The increase in mean air temperature is often associated with shifts in distribution, changes in phenology, and local extinctions. Other factors that only partially correlate with air temperature, like water shortage, may also contribute to the negative consequences of climate change. Although the effect of temperature on lizards’ ecophysiology is highly studied, many lizards are also at risks of increased water loss and dehydration, which are predicted to increase under climate change. Here we aimed for the first time to explore if lacertid lizards exposed to dehydration thermoregulate less precisely than hydrated lizards and if dehydrated lizards are less active, change the daily pattern of thermoregulation and balance water balance against thermoregulation. We exposed four lizard species with differences in the thermal preference to thermal gradients with or without a source of water. We measured preferred body temperatures, daily pattern of thermoregulation, and the use of space. Dehydration negatively affected thermoregulation in all investigated species. Dehydrated lizards reduced their preferred body temperature and showed a species-specific pattern of hourly change in thermal preference. Furthermore, they more frequently used the colder parts of the gradients and spent more time hidden. Lizards experiencing dehydration may suffer a reduction in survival and fitness because of poor thermoregulation. Similarly, they may spend more time hidden, waiting for more favourable weather conditions. Such inactivity may carry ecological costs especially in those regions that undergo either short or prolonged periods of droughts.

## Introduction

Changing climatic conditions over the past decades are affecting many animal species [1]. As a result, many taxa are experiencing range shifts [2], change in phenology [3], and local extinction [4] among other effects. However, climate change rate is not globally uniform [5], and some regions are experiencing a faster rise in temperature than others [6]. Furthermore, extreme climatic events, like heatwaves [7] and droughts [8] are becoming more common in some regions, like the Mediterranean basin [9]. Similarly, some species are more exposed to climate change either because they live in areas from which they cannot escape, like mountaintops [10], or because they are particularly vulnerable to the effect of rising temperatures and extreme climatic events, like many amphibians [11].

Reptiles depend strongly on the local external environment to maintain their body temperature within a well-defined range in which performance is optimal [12,13]. Lizards are receiving much attention in recent years since they are considered particularly vulnerable to abrupt changes in environmental temperature [14] that might reduce their activity budget [15] and lead to local extinctions [16]. Indeed, lizards appear to be particularly at risk, as some estimate that up to 20% of lizard species may be at risk of extinction by 2080 [16, but see [17] for the role of behaviour and habitat in modulating the effect of climate change in ectotherms]. The increase of air temperature is often used as the primary argument for explaining the adverse effect of climate change on animal populations, including lizards [16, 18]. However, there is only partial support for the increase in air temperature as being the primary cause of species extinction [19]. Other factors, which partially covary with air temperatures, like food and water availability, are likely among the primary variables determining the survival of a given population under climate change [20].

While the thermal ecology and physiology of lizards have been extensively studied for almost a century [13, 21, 22], much less is known on the negative consequences of water shortage on lizard behaviour, ecology, and conservation. At the individual level, for example, a recent experiment conducted in a confined environment showed that the rainfall regime affected lizards’ behaviour and activity. In particular, during dry periods lizards used shaded microhabitats more often, while they were more active in sunny microhabitats following rainfall [23]. In general, it seems that dehydrated lizards select for lower body temperatures, probably to contain water loss [24]. In temperate regions, water shortage or droughts may also affect lizards’ reproduction [25] and development, with yearlings exposed to dry condition showing lower growth and activity rates [26]. At the population level, lizards might be able to physiologically adjust their resistance to water loss in function of the availability of free-standing water [27].

To complicate the matter, a warmer and drier environment may affect lizards in complicated ways since the interactions between thermal and water balance, and how lizards react to optimize them are not clear. For example, a thermoregulating lizard may get dehydrated, which leads to an excessive concentration of plasma solutes [28]. On the other hand, while keeping suboptimal body temperature may prevent dehydration, it also leads to reduced locomotor and foraging performances [29,30]. Furthermore, geographically separated populations or closely related species might react differently to dehydration, reflecting local adaptation or plasticity [27]. Climate change may further blur the picture because some species or populations might be exposed to different patterns of increasing temperature and decreasing water availability. To what extent dehydrated lacertid lizards prioritise either thermoregulation or reducing water loss is still unknown and deserves further investigation.

Here, we aimed to assess the impact of dehydration on lizard thermoregulation. In particular, we quantified the effect of dehydration on lizard thermoregulation using four *Podarcis* species as a case study (*P*. *bocagei*, *P*. *carbonelli P*. *guadarramae*, and *P*. *virescens*). These lacertid species form a single clade that originated in the western Iberian Peninsula during the late Miocene [31], in an epoch during which the climate was more humid, cooler, and summer droughts were yet to come [32]. These species have been recently studied regarding thermal ecology [33] and resistance to water loss [34]. For instance, sympatric and sister taxa species differ in both thermal preference and resistance to water loss [33]. Further, these species show notable differences regarding habitat use [35] and morphology [36]. They also differ in their distribution patterns, with *P*. *bocagei* and *P*. *carbonelli* restricted to areas with Atlantic influence, while *P*. *guadarramae* and *P*. *virescens* tend to occupy more Mediterranean environments, although they may partially overlap either regionally or locally [37]. Their context of evolutionary history and ecology make *Podarcis* wall lizards an excellent system to investigate not only the impact of dehydration on thermoregulation, but also its variation across a clade of closely related, yet very distinctive species.

We formulate three testable hypotheses on the consequences of dehydration on thermal ecology of lizards. We expect that (1) dehydrated lizards will reduce their body temperature, to prevent or diminish further water loss, mainly by using more frequently cooler areas or spending more time hiding; that (2) thermoregulation will be more precise (the mean deviation from preferred temperature is smaller) when lizards have access to water (because lizards can optimize thermoregulation); and that (3) dehydrated lizards will be forced to physiologically trade-off body temperature and water retention. When water is not available, if some lizards will keep maintaining body temperatures close to the preferred ones, they will lose more water than those that will reduce their body temperature to avoid further water loss.

## Materials and methods

### Sampling and housing

Lizards were collected from two localities in Northern Portugal. In each, a pair of species can be found living in syntopy. We sampled *P*. *bocagei* and *P*. *guadarramae* near Moledo, a coastal region in Viana do Castelo province (41°50’ N, 8°52’ W), while we sampled *P*. *carbonelli* and *P*. *virescens* in Santa Maria da Feira, in Aveiro province (40°55’ N, 8°32’ W). Sampling spanned most of the activity season for these species in those locations (beginning of April–end of September). We captured only adult males with intact tails, to avoid reproductive status, body condition and ontogeny affecting thermal preferences [38]. We recognised each individual by means of photo-identification, to avoid pseudoreplication [39]. Before the experiment, we measured the snout-vent length (SVL) of each lizard to the nearest 0.01 mm with a digital calliper. We then left lizards undisturbed for 24 h in individual plastic cages (40×25×25 cm) and provided them with a source of water but with no food. After the first 24 h, we moved each lizard to a randomly chosen gradient (see below) for acclimation. We assumed that when the experiment started, lizards were fully hydrated, having had access to unlimited water during the previous 24 h.

### Preferred body temperatures

**Fig 1:**
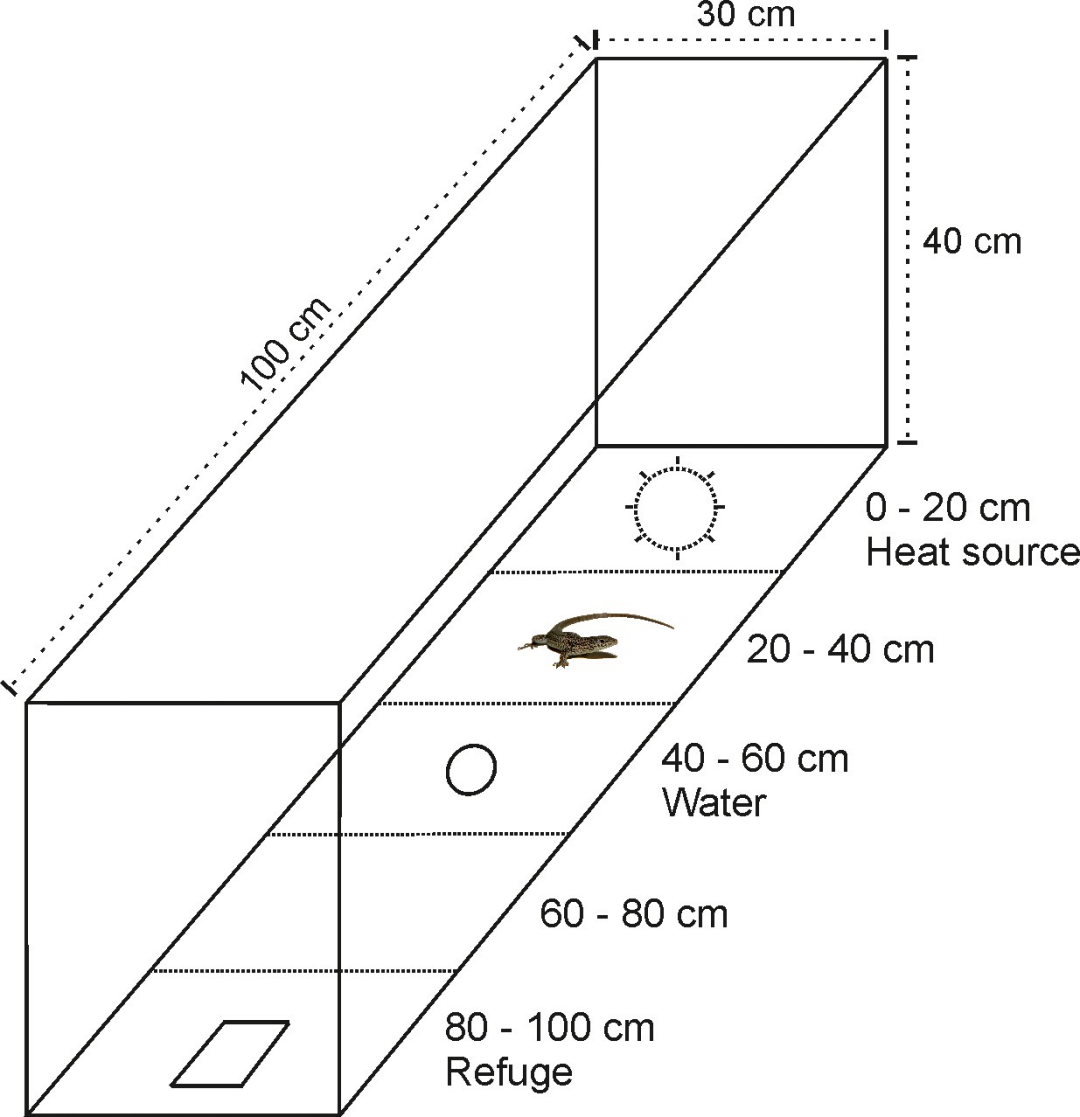
Scheme of the experimental setting used in this study. The five sections, 20 cm each, were only virtual and lizards had full access to every spot in the gradient. Just as an example, a lizard is depicted during thermoregulation in the most used section (see results). The two treatments differed only by the water container being full or empty.

We assessed the preferred body temperatures of lizards in linear thermal gradients following the method of [33]. Thermal gradients were set up in plastic containers (PVC; 100×30×40 cm; Fig 1) using sand as substrate (~1 cm deep). The gradients’ walls were opaque, and lizards could never see each other during the entire duration of the experiment. They were kept in a room at 20°C and illuminated by natural light through a window. A 150 W infrared lamp suspended above one end of each gradient created a range of temperatures from approximately 19 to 68°C (S3 Table). At the cooler end of each gradient, a small plastic box (13×8.5×5.5 cm) provided a refuge. We placed each lizard in a thermal gradient at 19.00 h when lights were off. The following morning, the infrared lights turned on at 08.00 h and turned off again at 19.00 h. On the first day, lizards were left undisturbed to explore the thermal gradient. At the end of the first day, we randomly emptied the water source (a plastic lid, ~13 ml of tap water) from half of the gradients and left it in the other half. On the next (second) day, the lamps were turned on during the same time span. Every hour between 9.30 and 18.30 h (10 measurements) we measured the skin temperature of each lizard by shooting a thermal picture using a FLIR T335 thermal camera (sensitivity: < 0.05°C; accuracy: ± 2% of the reading; IR image resolution: 320×240 pixels; FLIR Systems Inc., Wilsonville, Oregon, USA). For consistency, the same person (MS) shot all the photos. Skin temperatures were extracted from each IR picture using the *Spotmeter* function in FLIR Tools 5.12 (Copyright 2018 FLIR Systems, Inc; http://www.flir.com), selecting a spot in the last third of each lizards’ back. This position allowed us to avoid thermal heterogeneity in lizards’ body and correlate well with internal (cloacal) body temperature [33, 40]. At the end of the second day we reversed the treatments (the water was added to those gradients without it and removed from those gradients where it was present) and measured the hourly skin temperatures of each lizard during the following morning as described above.

### Spatial use of the gradients

Along with their main axis, gradients were divided into five sections of 20 cm each, visually identified by narrow strips of tape running along the walls (Fig 1). By dividing each gradient in this manner, we created five virtual sections (plus the refuge) characterized by different available temperatures, to assess whether lizards used the various areas differently depending on the treatment. Upon shooting each IR picture, we noted the position of the lizard inside the gradient, with respect to the five sections. If a lizard was inside the refuge, we noted this as a sixth position. By using such system, we were able to reference the spatial (linear) use of the gradients with a resolution of 20 cm and with one body temperature measurement every hour for each lizard.

### Body mass measurements

At the end of the second and third day (after taking the last thermal picture, at ~19.00 h), each lizard was briefly removed from its gradient and weighted to the nearest 0.1 mg using a precision balance (Sartorius M-Pact AX224, Sartorius AG, Goettingen, Germany). After the second day, lizards were released back into the gradients, while after the third day the experiment ended, and we left them for 24 h in individual boxes with water provided *ad libitum*. Subsequently, all lizards were released back to their respective site of capture. The difference in body weight between the control and experimental day (with or without access to water) was used to calculate the percentage of water loss (divided by the mass of the assumed fully hydrated lizard) for each individual. Since lizards did not have access to food during the entire period of the experiment, we ruled out that faeces (that are usually expelled in association with urine) might contribute to water loss. We confirmed such assumption by inspecting the gradients after the experiment. We were not able to find any residue that might suggest defecation.

### Statistical analysis

To quantify the effect of dehydration, species, time of the day and season on thermal preferences, we fitted a mixed-effects model with body temperature as the dependent variable, a triple interaction treatment × species × time plus a double interaction treatment × day of the season as the predictor. Because body temperature may vary non-linearly with time depending on the treatment, the variable “time” was fitted both as linear and quadratic terms, and the statistical difference of the resulting models was tested using log-likelihood ratio tests (*LR*) with the function *anova*.*lme*. The model with time as a quadratic term fitted the data better and hence was used for subsequent analysis (*LR* = 178.4, *P* < 0.0001) To account for repeated measures of the same individual and the possible temporal autocorrelation of the measurements, we included and tested for the effect of individual identity as random factor and repeated measures nested within individuals as autocorrelative structure. The difference between the models with and without random effect was significant (*LR* = 123.8, *P* < 0.0001) and, hence, random effects were retained. Instead, models with or without autocorrelation structure were not different (*LR* = 0.833, *P* = 0.36). Hence, repeated measures were considered as statistically independent in subsequent analysis. The arrangement of the refuge (water, refuge, lamp) may potentially biased the result. In particular, being the refuge in the cool part (as may be expected in nature for lizards living in temperate areas), dehydrated lizards seeking shelter may be forced to hide in a cool place. Hence, we re-run all analysis previously descripted removing the records of hiding lizards. By doing this, we were able to check if the treatment effect was still significant when active-only lizards were considered. The contribution of main factors and their interaction was assessed inspecting t-tables from models’ summary, and the effect size of the treatment was assessed using Cohen’s d [41]. We also fitted a linear model to test for a possible negative interaction between the amount of water loss and the resulting thermal preference (model form: percentage of water loss ~ mean body temperature (when dehydrated) × species, no random effect, each individual used only once). Percentages of water loss accounted for interindividual differences in body size and were normalised using square root (Shapiro-Wilk normality test (*W*) = 0.99, *P* = 0.55). We tested the significance of skewness in body temperature using the D’Agostino test. We tested for the decrease in thermoregulation precision by comparing the variances of body temperatures using F-tests. For both D’Agostino and F-tests, we accounted for repeated measures using the function *p*.*adjust*, set on 10 replicates and using the correction of [42]. Skewness values, both original and corrected, are reported in S1 Table. We tested if the use of the space was different under the two treatments using Chi-square tests with the R built-in function *chisq*.*test*. To test for interspecific differences in SVL and body mass, we used ANOVA. Statistical analysis was run in R version 3.5.0 [43]. We fit mixed-effects models with the *nlme* library [44], D’Agostino tests with the *moments* library [45], Cohen’s d effect sizes with the *effsize* library [46] and figures were produced using the library *ggplot2* [47]. Reported values represent mean ± standard deviation (SD) unless otherwise specified.

## Results

### Preferred body temperatures

**Fig 2:**
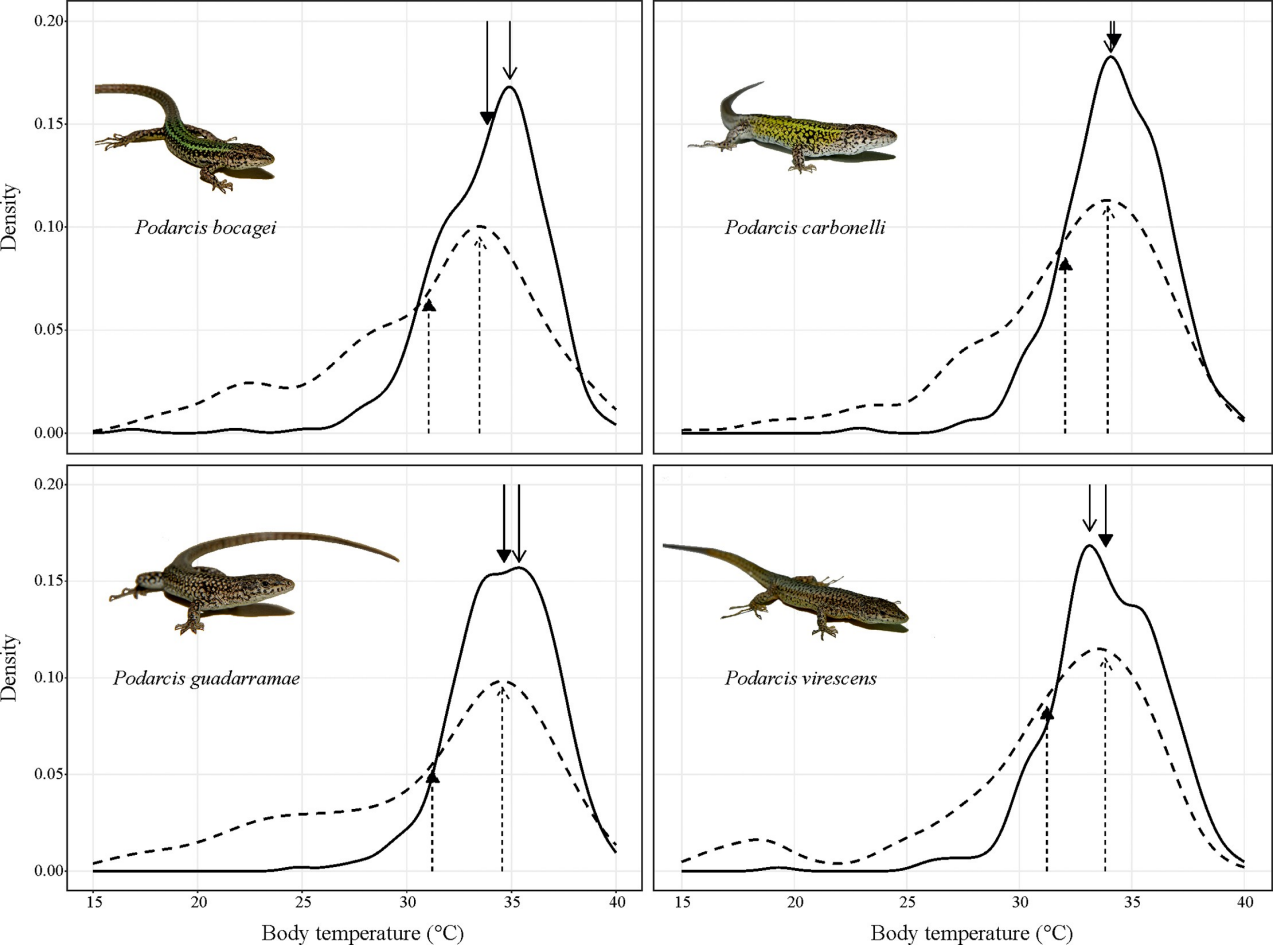
Distributions of the body temperatures selected by four lacertid species when water was either available in the gradient (solid lines) or not (dashed lines). Y-axis represents standardized (density) frequency of use for each body temperature. Closed arrows represent mean values; open arrows represent the modes (see Table 1 for the numerical values).

We tested 116 individuals, collecting a total of 2320 body temperature measurements (Table 1). When lizards did not have access to water, all species reduced significantly (*P*. *bocagei t-value* = 9.114, d.f. = 550, *P* < 0.0001; *P*. *carbonelli t-value* = 7.101, d.f. = 474, *P* < 0.0001; *P*. *guadarramae t-value* = 10.35, d.f. = 596, *P* < 0.0001; *P*. *virescens t-value* = 8.859, d.f. = 607, *P* < 0.0001; Fig 2) their mean body temperature, as well as the median and the mode of the distribution. When active-only lizards were considered, the treatment effect was still statistically significant (*t-value* = 2.77, d.f. = 1940, *P* = 0.0014). Similarly, we found no statistical support for a seasonal effect on thermal preferences when both treatments were considered (*t-value* = -0.001, d.f. = 2182, *P* = 0.56). Changes in curves distribution were more marked if mean values are considered (Table 1). The effect sizes (Cohen's d) ranged from moderate to large (Table 1), suggesting that one day without access to water is enough to negatively affect thermal preferences. Even though some species differed from each other in their preferred body temperature when water was available (S2 Table), such differences turned not significant when water was not available. As predicted, all species thermoregulated more precisely when fully hydrated (*P*. *bocagei F-test* = 0.292, d.f. = 289, *P* <0.0001; *P*. *carbonelli F-test* = 0.289, d.f. = 249, *P* <0.0001; *P*. *guadarramae F-test* = 0.186, d.f. = 299, *P* <0.0001; *P*. *virescens F-test* = 0.264, d.f. = 319, *P* <0.0001; Table 1). Furthermore, the effect of dehydration seemed mostly related to the statistical distribution of the data, rather than a shift in the preferred (mean) temperature (note the overlap of the open, dashed arrows in Fig 2). Indeed, body temperatures were left-skewed for all species and in both treatments (*P* < 0.01 in all cases, S1 Table). However, the skewness of the dehydrated lizards’ body temperature distribution curves increased, while peaks of distribution changed to a lesser extent (Table 1).

**Table 1:** Comparison of mean, median and interquartile range of the preferred body temperature of four lacertid (*Podarcis*) species when water was either available or not in the gradients. Values after ± represent the standard deviation. Paired Cohen’s d for the mean difference are calculated after Cohen (1988).

			Preferred	body	temperature (°C)				
Species	n	Treatment	Mean	Median	Interquartile range (75% - 25%)	Mean diff.	Median diff.	Mode diff.	Cohen’s d (mean diff.)
*P*. *bocagei*	29	Water	33.8 ± 2.7	34.2	35.6–32.2	2.8	1.9	1.5	0.83
		No Water	31.0 ± 5.0	32.3	34.6–28.2				
*P*. *carbonelli*	25	Water	34.2 ± 2.3	34.3	35.8–32.9	2.2	1.25	0.1	0.60
		No Water	32.0 ± 4.3	33.1	35.1–30.1				
*P*. *guadarramae*	30	Water	34.6 ± 2.4	34.9	36.3–33.2	3.4	1.8	0.8	0.79
		No Water	31.2 ± 5.5	33.1	35.2–27.7				
*P*. *virescens*	32	Water	33.8 ± 2.5	33.8	35.6–32.5	2.6	1.3	-0.7	0.68
		No Water	31.2 ± 4.9	32.5	34.6–29.6				

### Hourly pattern of body temperatures

**Fig 3:**
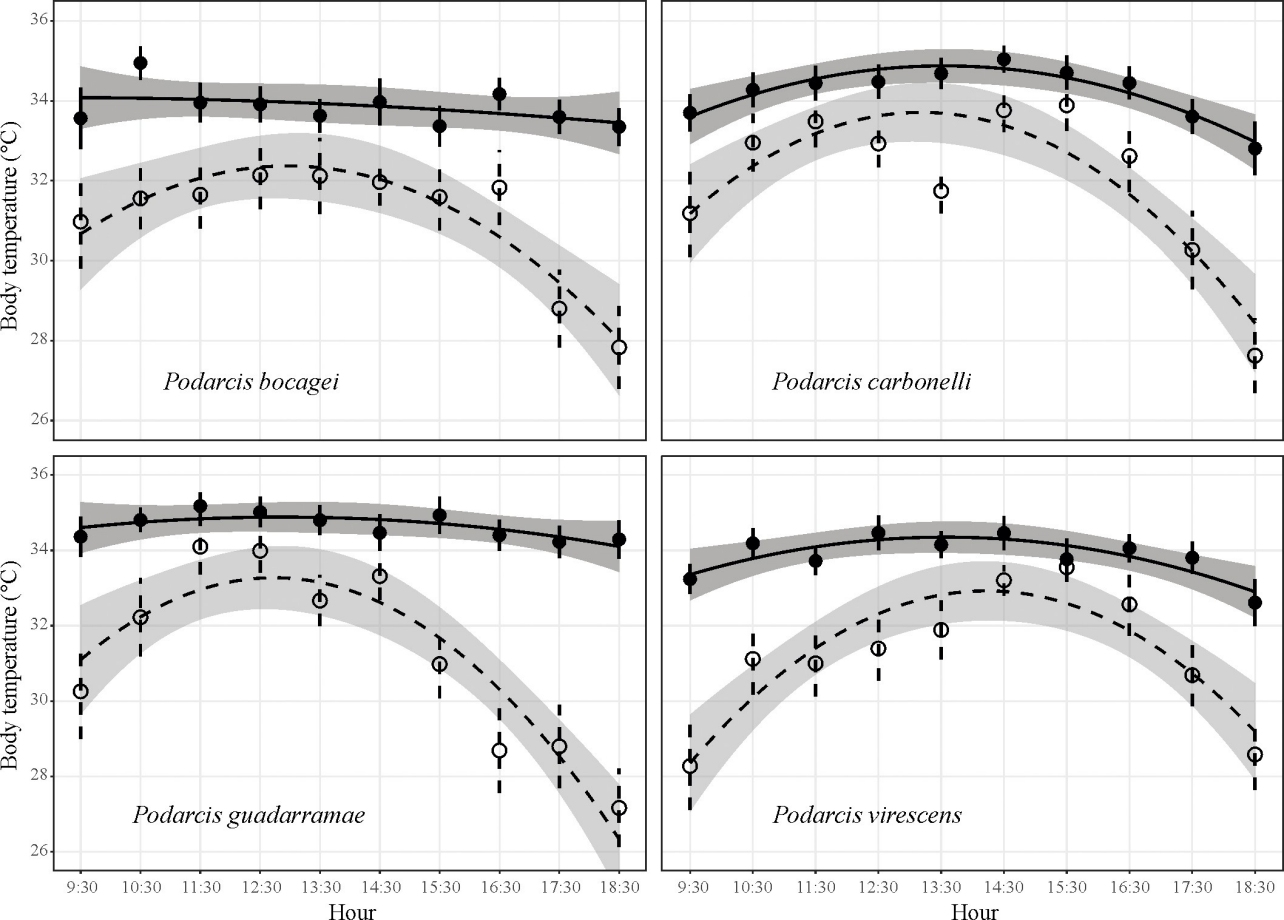
Hourly-based body temperature selected by the four lacertid species when water was either available in the gradient (solid lines and dots) or not (dashed lines and triangles). Vertical bars represent standard errors. Shaded areas represent 95% confidence intervals.

When the daily effect of dehydration on thermoregulation was considered, that is, the interaction treatment × time, and we found that its effect was significant for all species (*P*. *bocagei t-value* = 3.308, d.f. = 546, *P* = 0.001; *P*. *carbonelli t-value* = 2.769, d.f. = 470, *P* = 0.006; *P*. *guadarramae t-value* = 4.355, d.f. = 565, *P* < 0.0001; *P*. *virescens t-value* = 3.724, d.f. = 603, *P* = 0.0002). Such a result indicates not only that mean body temperature was different between treatments for all species, but also that such a difference changed along the day (Fig 3). More precisely, when water was not available, all species adopted a strong curvilinear strategy, with higher body temperatures selected during the central part of the day and lower temperature at the beginning and the end of the day. Still, even during the central part of the day, when dehydrated lizards thermoregulated the most, the difference between treatments was still significant at every time interval, as suggested by the 95% C.I. (Fig 3).

### Spatial use of the gradients

**Fig 4:**
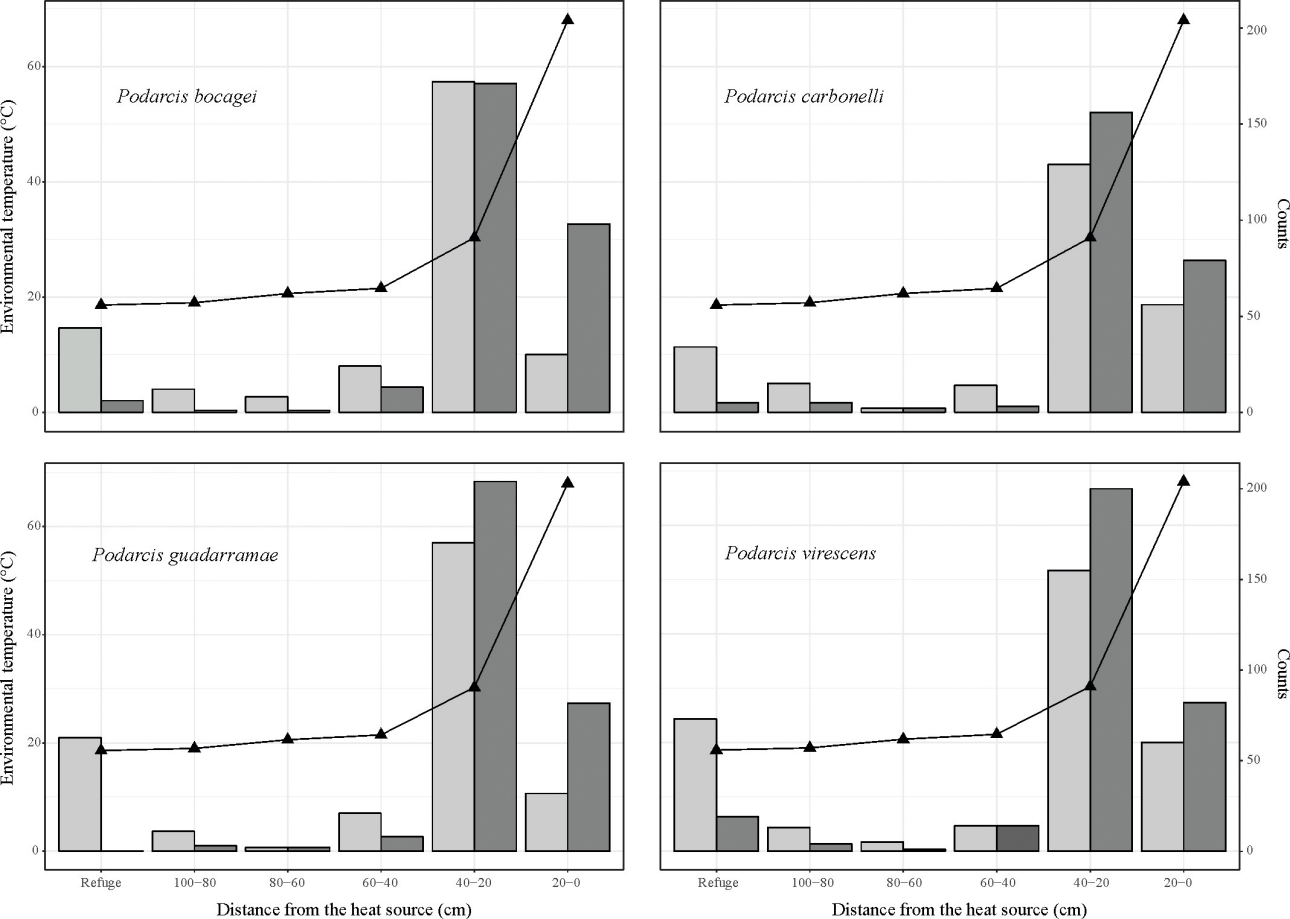
Spatial use of the gradients of four species of lacertid lizards. The x-axis represents the six areas into which each gradient was virtually divided. The leftmost part represents the refuge location, while the rightmost position was directly below the heating lamp. The left y-axis represents the available temperature in the gradient for each position (S3 Table and S1 Fig, Supporting Information for details on how it was estimated). The right y-axis represents the count of lizards for each position. Dark grey bars were counts for lizard when water was available in the gradient, while light grey bars represent lizards that had no access to water. The dark triangles represent the mean environmental temperature as measured by dataloggers for each segment of the gradient.

All species exploited the gradients differently depending on the treatment (*P*. *bocagei*: χ^2^ = 83.03, d.f. = 5, *P* < 0.0001; *P*. *carbonelli*: χ^2^ = 40.16, d.f. = 5, *P* < 0.0001; *P*. *guadarramae*: χ^2^ = 98.4, d.f. = 5, *P* < 0.0001; *P*. *virescens*: χ^2^ = 48.24, d.f. = 5, *P* < 0.0001). Lizards under both treatments spent most of their time within 20–40 cm from the lamp (Fig 1 and Fig 4). Such position offered the best spot for thermoregulation, with an environmental temperature of ~30°C (Fig 4; S3 Table and S1 Fig). The hottest spot in the gradient, directly under the lamp, was used more by hydrated lizards. On the contrary, the refuge, situated in the cool part of the gradient, was mainly used by dehydrated lizards (Fig 4). Lizards scarcely used intermediate sections, regardless of the treatment. Thus, they spent most of their time in the most favourable spot for thermoregulation, but when water was available, they also exploited the warmest area of the gradient since dehydration was not a limiting factor for thermoregulation. Conversely, lizards suffering from dehydration often sought refuge inside the shelter.

### The effect of dehydration on body mass

**Fig 5:**
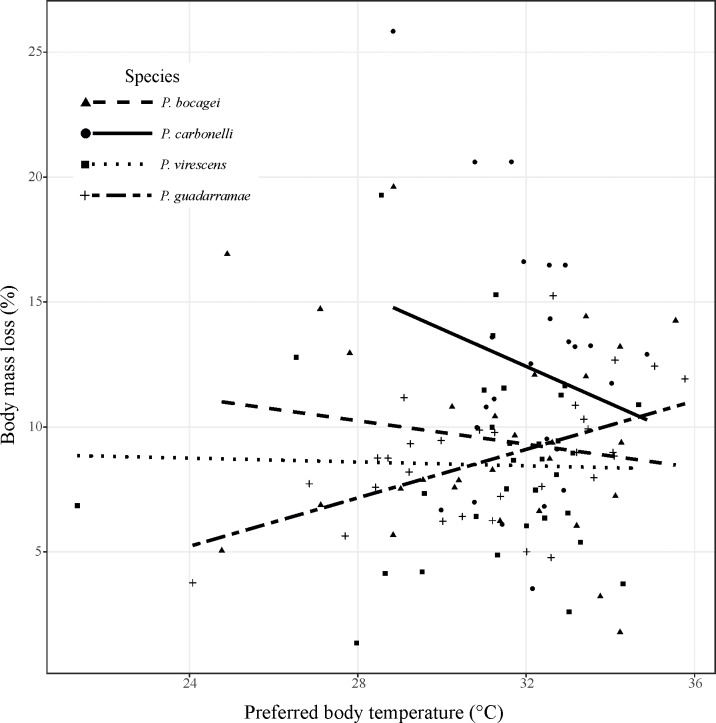
The species-specific relationship between preferred body temperature and water loss corrected for individual weights.

The four species of lizards differed in SVL (*F*_3,112_ = 8.771, P < 0.0001) and initial body mass (*F*_3,112_ = 16.6, P < 0.0001). After 24 h of thermoregulation without access to water, all species lost a significant amount of body mass (Table 2). At the end of the experiment, the most dehydrated species, relative to its body mass, was *P*. *carbonelli* (11.3%), while the less dehydrated was *P*. *virescens* (7.5%; Table 2). The effect size (Cohens’ d) was large for all species, suggesting that 24 h of water shortage are enough for imposing dehydration. The loss of body mass did not solely depend on body size. Indeed, even though the smallest species (*P*. *carbonelli*) suffered the most from dehydration, the biggest species (*P*. *bocagei*) was not the better in coping with water loss. Such result is reinforced by investigating the species-specific relationship linking water loss (as a proportion of body mass) and preferred temperature (Fig 5). Although this relationship is statistically non-significant for all species (P = 0.37), there is a significant difference in slope for *P*. *bocagei* and *P*. *guadarramae* (*t-value* = -1.985, *P* = 0.0497, d.f. = 108), a pair of sympatric species. This result suggests not only that individuals selecting higher body temperatures do not necessarily loose more water, but also that hydration level may elicit different thermoregulatory responses depending on the examined species.

**Table 2:** Body mass after 24 h either with or without access to water in the gradients. Differences in body mass are expressed both in grams and in percentages. Values are rounded to the second decimal value.

		Body mass (g)					
Species	SVL (mm)	With water	No water	Difference g (%)	*t*- value	*P*	Cohen’s d
*P*. *bocagei*	58.61 ± 3.68	4.10 ± 0.80	3.75 ± 0.74	0.35 (8.5)	10.58	< 0.0001	1.97
*P*. *carbonelli*	53.19 ± 4.40	2.74 ± 0.71	2.43 ± 0.65	0.31 (11.3)	10.31	< 0.0001	2.06
*P*. *guadarramae*	56.09 ± 3.33	3.33 ± 0.56	3.08 ± 0.55	0.25 (7.6)	19.21	< 0.0001	3.51
*P*. *virescens*	56.84 ± 4.30	3.34 ± 0.76	3.09 ± 0.71	0.25 (7.5)	9.08	< 0.0001	1.60

## Discussion

The study of thermoregulation, water balance and their interaction are of primary importance for a better understanding of how ectotherm species interact with their environment [12, 13]. Research on ecophysiology becomes even more compelling when one considers the evidence on how climate change is affecting ectotherms distribution and survival [1, 3, 10, 16]. Our results show that hydration state affects or interacts with thermoregulation. In the present case, dehydration led to reduced body temperature, increased skewness in body temperature distribution, changes in the hourly pattern of thermoregulation and the use of space. Hence, hydration state may interact with thermoregulation and possibly impose a physiological choice between thermoregulation and water balance.

### Effects of dehydration on preferred body temperature

Despite having the same thermal availability, dehydrated lizards were, on average, colder than hydrated ones and such difference may affect several aspects of lizards’ ecology. Lower body temperature may be detrimental to the ectotherms’ whole-individual performance as feeding, locomotion, reproduction, social interactions, as well as predator avoidance, all depend on the maintenance of optimal body temperature [22, 48, 49, 50]. In nature, dehydrated lizards may reduce activity, or under droughts, they may reduce activity to prevent dehydration. For example, it has been shown that lizards experiencing droughts are less active than those exposed to milder conditions [23]. As a consequence of reduced activity, lizards may face negative consequences concerning development, survival, and reproduction.

Here, dehydrated lizards also showed an increased skewness in body temperature distribution (Fig 2). Left-skewness in ectotherms body temperature is a well-known phenomenon that is expected for several reasons, like asymmetry in operative temperatures and thermal performance curves [51, 52, 53]. More broadly, skewness is an intrinsic feature of physiological processes, since they increase exponentially with temperature [54]. Recently, an extensive review of skewness in several desert lizard species concluded that left-skewness is common across geography and phylogeny, and does not correlate with body size or median body temperature [55]. Our results reinforce such a view, and the four species we tested reacted in a similar way to water deprivation (Fig 2). Heavily left-skewed body temperature distribution was mainly caused by the time they spent in unfavourabled thermal conditions (especially hiding inside the refuge). Thus, in the present case, dehydration directly affected the lizards’ thermoregulation processes, by limiting their ability to achieve and maintain their preferred body temperature.

### Change in daily pattern of thermoregulation

The second result of this study concerns the daily pattern of thermoregulation. Lizards were able to keep high body temperature throughout the day as long as dehydration was not a limiting factor. The same individuals, however, selected lower body temperatures when dehydrated. All species kept lower body temperatures at the beginning and the end of the day, while they raised their body temperature during the middle part of the day. Even at their maximum, body temperatures of dehydrated lizards were always lower than those of hydrated lizards. Since lizards spent only one day in the lab before experimentation (with access to natural light), they likely remained synchronised with circadian rhythm. This result could thus reflect an attempt to thermoregulate only during the hottest part of the day when perhaps the probability of finding preys (and thus replenish water) is the highest. Such a strategy would be related to the concept of *hours in restriction of activity* [16], postulating that lizards exposed to extremely high environmental temperature will be forced to reduce their activity to dawn and sunset. Our results, however, suggest that lizards tried to maximise thermoregulation during the central part of the day. For ectotherms living in temperate areas experiencing summer droughts, this strategy may conflict with excessively high environmental temperature. Indeed, during summer, many species of European lizards shift to a bimodal pattern of activity [56, 57]. We thus suggest that, on a daily basis, thermoregulation and water balance may occasionally conflict, likely because of the complex interaction between foraging behaviour and the physiological balance of heat and water.

### Dehydration constrains the use of space

In the gradients, lizards had access to a wide range of available temperatures and a refuge. In both treatments, lizards spent most of their time in the position that offered the best spot for thermoregulation. The main difference was reflected in the use of gradient extremes, hot and cold. Dehydrated lizards were more prone to use the refuge, while fully hydrated ones spent more time under the lamp. A similar result was found in *Sceloporus* lizards, which buried more often when dehydrated [24]. Such behavioural differences may result from the attempt of dehydrated lizards to both thermoregulate and avoid water loss. Indeed, even under dehydration, lizards still selected the best spot for thermoregulation, but it is likely that they evaluated the water loss more carefully and retreated more often. An alternative explanation would be that dehydrated lizards had lower temperature because were searching for water instead of thermoregulating. However, our results do not support such a hypothesis, since dehydrated lizards most of the time selected the same spot fully hydrated ones or retreated. Interestingly, when water loss was not a constraint, lizards used the hottest spot in the gradients more often. This behaviour may maximise the heat intake per a given unit of time in a situation in which individuals could indefinitely replenish the water lost through evaporation. This offers a hypothesis testable in the field. Lizards subjected to higher water stress should refrain from reaching a high body temperature or from maintaining it for prolonged periods of time. Other ecological consequences of a reduced availability of suitable habitats may involve increasing intraspecific competition and limited opportunities to find food items and mates.

### Interspecific differences

Dehydration elicited behavioural responses that appear to be qualitatively similar in the four species we tested. All species decreased the mean body temperature, levelling on similar values. Even the sympatric pair *P*. *bocagei* and *P*. *guadarramae*, that differed in preferred body temperature when hydrated (S2 Table), showed no difference under dehydration. Similarly, all species showed a linear or nearly linear trend in daily thermoregulation when fully hydrated but switched to a parabolic trend when dehydrated and retreated more often in the refuge when dehydrated (Fig 3). However, some species differed in the slope of the regression between the preferred temperature and the body mass lost (Fig 5). In particular, *P*. *bocagei* and *P*. *guadarramae* showed a significant difference in their slopes, suggesting that the negative effects of dehydration on thermoregulation are species-specific, and may differ even in sympatric sister species. Indeed, while in *P*. *guadarramae* the loss of water was positively associated with preferred body temperature, the reverse was true for *P*. *bocagei*. Interestingly, the other pair of sister species tested, *P*. *carbonelli* and *P*. *virescens*, showed no differences both in thermal preferences and in the regression slope between the preferred temperature and the body mass lost, despite the fact that the distributions of these two species overlap only over a narrow strip along the costal Portugal [37]. These results reinforce recent findings suggesting that in sympatric lacertid species water balance may often be prioritized, with a potential detrimental effect on thermoregulation. However, the daily and seasonal dynamics may greatly differ depending on the species and environments considered [34, 58, 59].

### Thermoregulation is compromised by dehydration

Combining the evidence collected, it appears that hydration level interacts with thermoregulatory behaviour by inducing an adjustment in the distribution of the selected body temperatures. When lizards had full access to water and dehydration posed no restrictions, they optimised thermoregulation, kept a constant body temperature throughout the day, and basked even in the hottest part of the gradients. Optimizing water balance would instead imply refraining from activity and retreating to the coolest spot available since in reptiles both activity level and body temperature determine the rate of water loss [60]. Dehydrated lizards, however, did not cease activity, nor selected the lowest body temperature possible. Instead, they employed a mixed-strategy, thermoregulating at lower temperatures and more often seeking shelter in the refuge. Why dehydrated lizards did not maximize water balance? We speculate that total inactivity would not represent a solution for dehydrated lizards. Instead, a mixed strategy of thermoregulation and retreat may offer more chances to find prey items or free-standing water, and other lizard species show increased activity when rainfall occurs after extended dry periods [61]. Such a strategy, however, may be inadequate under chronic water shortage, such as during European summer droughts [9].

### Dehydration and thermal preferences estimation

Our results also bear some implications for the quantification of thermal preferences under laboratory conditions, even though it should be considered that our experiment lasted overall three days—with one day of water deprivation—instead of the typical single one. Usually, lizards being tested in thermal gradients are not provided with water, because some definitions of thermal gradients stated that they should provide an environment free from ecological costs and constraints [62, 63]. Few authors justified the choice to not provide water if the studied species is insensitive to water loss in the short term [64]. We believe that such awareness is still rare and, given the results obtained here, we advise for caution. If the thermal preferences of the studied species are entirely unknown, or if its ecology, physiology, or distribution suggest that it may be sensitive to water loss, providing water in the gradients should be considered. Otherwise, a waterless gradient would violate the assumption that it should be cost-free environment. Furthermore, collected data on thermal preferences, although internally coherent, might be left-skewed due to the interaction between thermal preference and dehydration, and might underestimate preferred temperature.

Some practical aspects may limit the insights that this study provides about the effect of dehydration on thermoregulation. Firstly, we assumed that lizards were fully hydrated at the beginning of the experiment since they had had full access to an unlimited source of water. However, we cannot prove this assumption. Thus, our estimate of water loss reflects the percentage of body mass that each lizard lost under dehydration. Secondly, the effects of dehydration, as quantified in laboratory conditions, may be different under natural conditions. For instance, air temperature and humidity were kept constant in this study and did not fluctuated as they do naturally under circadian rhythms. Thirdly, we focused on the short-term effects of dehydration, and the results of this study cannot be readily extrapolated to infer chronic (e.g., seasonal or annual) stress. Finally, for practical reasons, we investigated the use of space in a confined setting that by necessity is an oversimplification of a natural environment. We believe, however, that such limitations did not prevent us to accurately measure how dehydration affects thermoregulation in lizards.

## Conclusions

The present study shows that dehydration may negatively affect thermoregulation in lacertid lizards by forcing them to select body temperatures lower than the preferred ones. Poor thermoregulation may have profound consequences on survival, fitness, and life-history traits in ectotherms [22, 25, 26]. We showed here that dehydrated lizards often selected lower body temperatures more than the preferred ones, thermoregulate less precisely, and change the use of space. Available data on recent climatic scenarios, as well as predictions on future ones, indicate not only a trend in rising temperatures [65] but also an increase in the frequency of extreme heatwaves and droughts, at least for specific regions, like Europe [7, 8]. Temperate reptiles, like the lacertid lizards used in the present study, may be particularly vulnerable to such shortages in water availability since they appear to cope poorly with such climatic extremes [23]. Future research should pose considerable attention on the short and long-term effect that water shortage may impose on wild populations of ectotherms.

## Supporting information

S1 Fig Environmental temperature in the gradients. Boxplot of environmental temperatures available in the gradients as measured with dataloggers. At the end of the experiment, we randomly chose two gradients. In each, we placed six iButton Thermochron (Model DS1923; Maxim Integrated Products, Sunnyvale, CA, USA). Each datalogger was positioned in the middle of each of the five sections into which the gradients were virtually divided. The sixth datalogger was placed inside the refuge. Each datalogger was 20 cm away from the next, except the first one, that was close (~ 7 cm) to the one positioned inside the refuge. Dataloggers were set to record temperature every 30 mins and were retrieved after seven days. We merged the data from the two gradients and of the six positions to create a distribution of temperature for each section of the gradients.
(TIF)

S1 Table Skewness in preferred body temperature in four species of *Podarcis* lizards subjected to two treatments. In “water” treatment, individuals had had full access to a water source throughout the experiment. On the contrary, a water source was not available during the “no water” treatment. Skewness was calculated for both treatment, and its statistical significance tested with D’Agostino test. Original P-values are reported as well as corrected ones for multiple individual measurements (for each lizard body temperature was measured ten times).
(PDF)

S2 Table Interspecific differences in reaction to dehydration. Even though some of the tested species differ in their thermal preference when fully hydrated, their body temperature did not differ when dehydrated. Such a result suggests that, despite having different thermal ecology under optimal conditions, dehydration likely impose similar physiological constrains on all tested species, forcing them to converge on comparable, lower body temperatures.
(PDF)

S3 Table Environmental temperature in the gradients. Mean environmental temperatures ± standard deviation for each section of the gradient.
(PDF)
