# Supplementary figures and images for: Dehydration constrains thermoregulation and space use in lizards
Source: PLoS One. 2019 Jul 25;14(7):e0220384. doi: 10.1371/journal.pone.0220384 (PMC6657907; doi:10.1371/journal.pone.0220384)

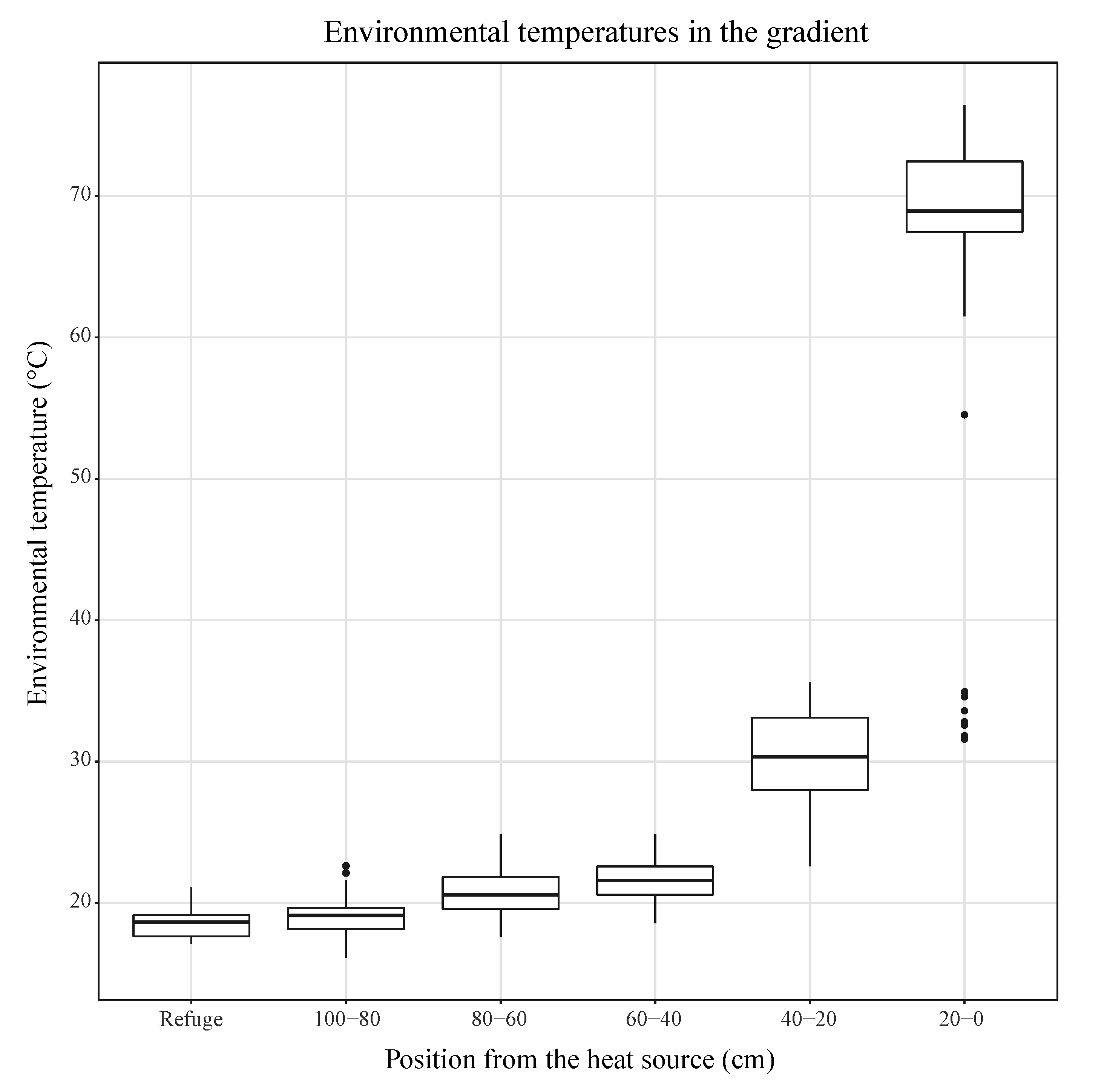

Supplement: S1 Fig — Boxplot of environmental temperatures available in the gradients as measured with dataloggers. At the end of the experiment, we randomly chose two gradients. In each, we placed six iButton Thermochron (Model DS1923; Maxim Integrated Products, Sunnyvale, CA, USA). Each datalogger was positioned in the middle of each of the five sections into which the gradients were virtually divided. The sixth datalogger was placed inside the refuge. Each datalogger was 20 cm away from the next, except the first one, that was close (~ 7 cm) to the one positioned inside the refuge. Dataloggers were set to record temperature every 30 mins and were retrieved after seven days. We merged the data from the two gradients and of the six positions to create a distribution of temperature for each section of the gradients. (TIF) [file pone.0220384.s001.tif]
