# Supplementary material for: Dehydration constrains thermoregulation and space use in lizards
Source: PLoS One. 2019 Jul 25;14(7):e0220384. doi: 10.1371/journal.pone.0220384 (PMC6657907; doi:10.1371/journal.pone.0220384)
Supplement: S1 Table — In “water” treatment, individuals had had full access to a water source throughout the experiment. On the contrary, a water source was not available during the “no water” treatment. Skewness was calculated for both treatment, and its statistical significance tested with D’Agostino test. Original P-values are reported as well as corrected ones for multiple individual measurements (for each lizard body temperature was measured ten times). (PDF) [file pone.0220384.s002.pdf]

S1 Table. Skewness in preferred body temperature in four species of *Podarcis* lizards subjected to two treatments.

Species	Treatment	Skewness	Z-value	*P* original	*P* adjusted
P. bocagei	Water	-0.79	-5.053	2.176×10^{-07}	6.373×10^{-06}
	No Water	-0.78	-4.967	2.176×10^{-07}	2.176×10^{-07}
P. carbonelli	Water	-0.61	-3.791	7.507×10^{-05}	0.0022
	No Water	-1.12	-6.146	3.974×10^{-10}	1.163×10^{-08}
P. guadarramae	Water	-0.52	-3.580	0.0002	0.0050
	No Water	-0.90	-5.694	6.219×10^{-09}	1.822×10^{-07}
P. virescens	Water	-0.82	-5.423	2.929×10^{-08}	8.579×10^{-07}
	No Water	-1.42	-8.140	2.22×10^{-16}	6.502×10^{-15}
