# Supplementary material for: Dehydration constrains thermoregulation and space use in lizards
Source: PLoS One. 2019 Jul 25;14(7):e0220384. doi: 10.1371/journal.pone.0220384 (PMC6657907; doi:10.1371/journal.pone.0220384)
Supplement: S2 Table — Even though some of the tested species differ in their thermal preference when fully hydrated, their body temperature did not differ when dehydrated. Such a result suggests that, despite having different thermal ecology under optimal conditions, dehydration likely impose similar physiological constrains on all tested species, forcing them to converge on comparable, lower body temperatures. (PDF) [file pone.0220384.s003.pdf]

S2 Table. Interspecific differences in reaction to dehydration.

Pair comparison	Mean difference (°C)	*t*-value	*P*
P. bocagei – P. carbonelli	-0.37	-1.207	0.4548
P. bocagei – P. guadarramae	**-0.80**	-3.530	**0.0021**
P. bocagei – P. virescens	0.00	0.520	0.6033
P. carbonelli – P. guadarramae	-0.43	-2.177	0.1176
P. carbonelli – P. virescens	0.37	1.733	0.249
P. guadarramae – P. virescens	**0.80**	4.141	**0.0002**
