# Supplementary material for: Dehydration constrains thermoregulation and space use in lizards
Source: PLoS One. 2019 Jul 25;14(7):e0220384. doi: 10.1371/journal.pone.0220384 (PMC6657907; doi:10.1371/journal.pone.0220384)
Supplement: S3 Table — Mean environmental temperatures ± standard deviation for each section of the gradient. (PDF) [file pone.0220384.s004.pdf]

S3 Table. Environmental temperature in the gradients.

Position (from the heat source, cm)	n	Mean ± SD
0 - 20	294	68.03 ± 8.35
20 - 40	294	30.27 ± 3.23
40 - 60	294	21.47 ± 1.23
60 - 80	294	20.63 ± 1.63
80 - 100	294	19.05 ± 1.26
Refuge	294	18.62 ± 0.95
